# From crisis to consequence: a first-hand look at the 2024 Valencia floods, climate disasters and psychiatric response

**DOI:** 10.1192/j.eurpsy.2026.10384

**Published:** 2026-07-31

**Authors:** J. P. Carrasco, A. Cerame

**Affiliations:** 1 Psychiatry, Hospital Provincial Castellón, Castellón; 2 Salud Mental, Servicio Madrileño de Salud, Madrid, Spain

## Abstract

**Background:**

Extreme weather events associated with climate change, such as the recent DANA (Depresión Aislada en Niveles Altos) floods in the Valencian Community, pose significant challenges not only to physical infrastructure but also to population mental health. Understanding how mental health professionals perceive and respond to such crises is essential to inform preparedness and policy.

**Methods:**

We conducted a qualitative study using a focus group methodology during the annual conference of the Asociación Española de Neuropsiquiatría (AEN) held in Valencia. Participants included psychiatrists, psychologists, and other mental health professionals directly or indirectly involved in the response to the DANA-related crisis. The discussion explored perceptions of institutional preparedness, political response, clinical impact, and preferred models of psychosocial intervention. Data were analyzed thematically.

**Results:**

Participants highlighted that the magnitude of psychosocial impact was not solely attributable to the natural disaster itself, but was significantly shaped by broader structural factors. Climate change was identified as an escalating contextual determinant increasing the frequency and severity of such events. Additionally, insufficient institutional preparedness and a perceived lack of timely and coordinated political response during the acute phase of the crisis were considered key contributors to the overall impact on affected communities.

Regarding professional response, participants valued most positively the opportunity to integrate into first-line community support and relief networks during the emergency. Strengthening pre-existing community mental health services and creating flexible psychosocial support units embedded within affected territories were perceived as more effective and sustainable strategies than the deployment of highly specialized trauma-focused units. While trauma-informed care was recognized as important, there was consensus that community-based, network-oriented interventions better addressed the complex and evolving needs of the population.

**Conclusions:**

The findings underscore the need to frame mental health responses to climate-related disasters within a broader socio-political and structural context. Strengthening community mental health networks, improving institutional preparedness, and integrating mental health professionals into coordinated first-line response systems may enhance resilience and reduce long-term psychosocial impact in future climate emergencies.

**Disclosure of Interest:**

None Declared

